# Recent advances in therapeutic targets identification and development of treatment strategies towards *Pseudomonas aeruginosa* infections

**DOI:** 10.1186/s12866-023-02832-x

**Published:** 2023-03-30

**Authors:** Daniel Ruben Akiola Sanya, Djamila Onésime, Grazia Vizzarro, Nicolas Jacquier

**Affiliations:** 1 Université Paris-Saclay, INRAE, AgroParisTech, Micalis Institute, Jouy-en-Josas, 78350 France; 2 Institute of Microbiology, University Hospital and University of Lausanne, Lausanne, 1011 Switzerland; 3 Present Address: Laboratory of Molecular Microbiology, Global Health Institute, School of Life Sciences, Station 19, EPFL-SV-UPBLO, Ecole Polytechnique Fédérale de Lausanne (EPFL), Lausanne, 1015 Switzerland

**Keywords:** Antibiotics, Antimicrobial agents, *Pseudomonas aeruginosa*, Biofilm, Resistance, Diagnostics, Vaccine

## Abstract

The opportunistic human pathogen *Pseudomonas aeruginosa* is the causal agent of a wide variety of infections. This non-fermentative Gram-negative bacillus can colonize zones where the skin barrier is weakened, such as wounds or burns. It also causes infections of the urinary tract, respiratory system or bloodstream. *P. aeruginosa* infections are common in hospitalized patients for which multidrug-resistant, respectively extensively drug-resistant isolates can be a strong contributor to a high rate of in-hospital mortality. Moreover, chronic respiratory system infections of cystic fibrosis patients are especially concerning, since very tedious to treat. *P. aeruginosa* exploits diverse cell-associated and secreted virulence factors, which play essential roles in its pathogenesis. Those factors encompass carbohydrate-binding proteins, quorum sensing that monitor the production of extracellular products, genes conferring extensive drug resistance, and a secretion system to deliver effectors to kill competitors or subvert host essential functions. In this article, we highlight recent advances in the understanding of *P. aeruginosa* pathogenicity and virulence as well as efforts for the identification of new drug targets and the development of new therapeutic strategies against *P. aeruginosa* infections. These recent advances provide innovative and promising strategies to circumvent infection caused by this important human pathogen.

## Background

*Pseudomonas aeruginosa* is a heterotrophic, motile, Gram-negative bacterium, which clinical isolates can be highly diverse regarding their genetic backgrounds and antimicrobial resistance profiles. *P. aeruginosa* is an opportunistic pathogen causing nosocomial and ventilator-associated infections with a high mortality rate [1]. Infections by this pathogen are of especially high importance for immunocompromised and cystic fibrosis (CF) patients. CF is a genetic disorder caused by mutations in the cystic fibrosis transmembrane conductance regulator that provoke an abnormal thickening of mucus, impaired ciliary function, and weakening of pulmonary immune response. Those alterations create an ideal microenvironment for lung infection by *P. aeruginosa* [2].

*P. aeruginosa* is intrinsically resistant to many antimicrobials, having a limited outer membrane permeability, expressing a wide variety of efflux pumps and producing AmpC, an inducible cephalosporinase. It can quickly develop antibiotic resistance through chromosomal mutations or horizontal gene acquisition. For example, the hypermutability of *pmrB* (10^3^–10^4^ times the background mutation rate) in PmrAB regulatory system facilitates a rapid adaptation to colistin [3]. Antibiotics having to cross the cell wall to reach their targets, such as aminoglycosides or polymyxins, have limited diffusion through *P. aeruginosa* cell envelope due to restricted permeability of the outer membrane and, in some cases, to the overexpression of outer membrane proteins, which restrain interaction of lipopolysaccharides (LPS) with the antimicrobial agents [4]. Furthermore, small hydrophilic antibiotics such as ß-lactams and quinolones that are able to cross the outer membrane through porins are expelled by efflux pumps [5]. *P. aeruginosa* can get further resistance by overexpression of AmpC, acquisition of mutations causing AmpC hyperactivity or through modification of the antimicrobial target structures [6]. In addition, horizontal gene transfer plays an important role, allowing the acquisition of resistance genes towards carbapenems [7] or quinolones (*qnrB*, *qnrA*, and *qnrS*), among others [8].

The emergence of multidrug-resistant (MDR) *P. aeruginosa* isolates has become a public health threat worldwide as infection by these isolates restricts treatment options and augments morbidity and mortality [1]. The emergence of carbapenem resistance in *P. aeruginosa* is particularly concerning, predominantly among critically ill patients, since carbapenems are an important treatment option against drug-resistant Gram-negative bacteria. *P. aeruginosa* is thus listed by World Health Organization as a critical priority pathogen urgently requiring novel treatment options [9].

*P. aeruginosa* does not only develop resistance, but can also acquire antibiotic tolerance through formation of biofilms, which are complex clusters of bacteria attached to a surface and embedded in a self-produced matrix. Biofilm formation by *P. aeruginosa* can lead to the development of nosocomial urinary tract infections (UTIs), catheter-associated UTIs, surgical site infections, infections in burn-wound patients and bloodstream infections, all associated with high rates of morbidity and mortality [10]. Bacteria entrapped in biofilms can be up to 1000-fold more tolerant to antibiotics than free-living bacteria, making treatment of such infections highly tedious [11]. In addition, some strains of *P. aeruginosa* are hyperbiofilm-forming, exhibiting some rugose small colony variants (RSCVs), developing biofilm aggregates surrounded by an extracellular matrix containing fragmented extracellular DNA and responsible for persistent infections, resistance to disruption by DNaseI and enhancement of biofilm formation [12].

Despite being an important human pathogen, *P. aeruginosa* is also widely present in the environment. Interestingly, some strains can have beneficial effects. *P. aeruginosa* is frequently found in sediments from ditches and tributaries and it displays an important role in nitrogen cycling in agriculture through its utilization of nitrate and urea [13]. Moreover, *P. aeruginosa* shows potential applications in industrial processes. For example, the strain *P. aeruginosa* ISJ14 degrades low-density polyethylene with no side effects on health or the environment, illustrating the potential of *P. aeruginosa* in waste processing [14]. Other applications were developed: Rilda et al. took advantage of *P. aeruginosa* antibacterial features in the construction of anti-bacterial textile fibres based on ZnO–TiO_2_ nanorods template [15].

In this review, we discuss recent discoveries regarding pathogenesis mechanisms of *P. aeruginosa* and how these discoveries may lead to the identification of novel drug targets. We then highlight recent breakthroughs in the development of antimicrobial agents targeting *P. aeruginosa*, and showcase the potential of vaccination as an alternative to strengthen host immune responses and counteract antibiotic tolerance or resistance from this bacterial pathogen.

## Main text

### Pathogenesis of *Pseudomonas aeruginosa*

*P. aeruginosa* mainly causes hospital-acquired respiratory infections, but can also infect wounds, surgical sites, urinary tract and even provoke bacteremia [1]. An essential step for *P. aeruginosa* pathogenesis is its adhesion to its host. This is mediated by flagella and pili that induce attachment to epithelial cells via respiratory mucins and glycolipid asialoGM1 [16]. Several host factors are important for efficient *P. aeruginosa* binding to host, including carbohydrate-binding proteins (lectins). It was recently shown that a fucose-binding lectin, LecB, plays an important role in the high-affinity host-cell binding of *P. aeruginosa* [17]. LecB contains a carbohydrate-binding site composed of two closely located calcium ions, which create a bridge between the ligand and the protein. The high-affinity binding of LecB relies on a low-barrier hydrogen bond, cooperative rings of hydrogen bonds, coordination contacts leading to a unique charge delocalization, and the mobility of trapped water molecules [17]. Importantly, adhesion of *P. aeruginosa* to respiratory epithelial cells is further increased in CF patients. Indeed, *P. aeruginosa* adheres to the CF epithelium at early stages of infection in a process that is facilitated by the apical overexpression of the Vav3 protein in CF airway epithelial cells. This protein stimulates β1 integrin and fibronectin production at the luminal side of epithelial cells, leading to enhanced *P. aeruginosa* adhesion [18].

Adhesion mechanisms are not the only contributors to the pathogenesis of *P. aeruginosa*. Interestingly, some clinical *P. aeruginosa* isolates found in diverse infected human body sites, encode a defective *lasR*, constitutively expressing a biofilm-adapted transcriptional profile without a need for environmental stimulus. The *lasR* gene encodes the major quorum-sensing (QS) regulator LasR and defective *lasR* genotype is suggested to contribute to the success of these clinical *P. aeruginosa* isolates [19].

Additional connections between the QS of *P. aeruginosa* and the regulation of its virulence have been provided. *P. aeruginosa* QS mechanism is initiated by the production of cell-to-cell signals, so-called quorum sensing autoinducer (QSAI) molecules [20]. The massive delivery of QSAI molecules by approximately two thousands cells is essential for *P. aeruginosa* aggregates to activate QS [21]. Several QS circuits are essential in this process: LasI and RhlI produce quorum sensing autoinducers (QSAI), which are sensed by LasR and RhlR. Binding of QSAI to LasR and RhlR regulate expression of large sets of genes, including virulence factors such as pyocyanin or rhamnolipids, among others [22]. Moreover, RhlS, a QS small noncoding RNA, triggers the translation of the global virulence regulator Vfr, the interacting partner of the RNA-binding protein Hfq [23].

During biofilm formation, *P. aeruginosa* secretes several exopolysaccharides (EPS), including alginate, Psl, and Pel. Detailed structure and expression regulation of these EPSs are reviewed elsewhere [24]. Psl encourages and sustains airway microbial community development, being important for attachment of bacteria to abiotic and biotic surfaces. Pel is a positively charged EPS, which plays an important role in cell to cell interactions in biofilm. Alginate is a high molecular weight polymer and is involved in the structural stability of biofilms and protects it from dehydration. These EPSs are also implicated in the development of microbial communities in the respiratory tract. For example, in the presence of *P. aeruginosa* environmental and clinical isolates, *Streptococcus salivarius* employs the maltose-binding surface protein MalE to interact with the *P. aeruginosa* Psl. This interaction initiates and sustains *S. salivarius* biofilm formation within the CF lung [25]. Bacterial biofilms still remain a challenge to the healthcare system due to their resilience against antimicrobial agents and immune system. Moreover, mechanisms involved in biofilm formation and/or biofilm antimicrobial tolerance in *P. aeruginosa* are only partially understood, making research in this field highly relevant. Recently, the small hypothetical protein encoded by the gene PA2146 and conserved in ɣ-proteobacteria has been shown to regulate biofilm architecture and antimicrobial tolerance of *P. aeruginosa* PAO1. The deletion of this gene did not impact growth rate of planktonic cells or minimal inhibitory concentrations of antibiotics against them, but seriously impaired *P. aeruginosa* PAO1 biofilm architecture and antimicrobial tolerance in presence of tobramycin [26].

It has been recently established that *P. aeruginosa* strains presenting a mucoid phenotype and playing a major role in the pathogenicity of *P. aeruginosa* towards CF patients [27] secrete two types of QSAI molecules: C4-HSL and PQS [28]. The C4-HSL molecule interacts with EPS solely through Van der Walls interactions and is thermodynamically stable within the vicinity of the EPS. The PQS molecule forms thermodynamically stable ionic complexes with EPS-bound Ca^2+^ and establishes a hydrogen bond with a single EPS chain [28]. Iron/siderophore acquisition systems also significantly contribute to virulence-related phenotype, such as biofilm formation and enhance the pathogenesis of hypervirulent *P. aeruginosa* in wound infection isolates [29]. In addition, *P. aeruginosa* produces virulence factors, such as phenazine, pyocyanin, pyoverdin, and rhamnolipid, regulated by multiple QS-pathways, to trigger pathogenicity [30].

Beside adhesion and biofilm formation, *P. aeruginosa* also needs to escape the immune response to efficiently infect its host. There has been tremendous interest in understanding the molecular mechanisms involved in the injection of effector proteins into eukaryotic host cells via the *P. aeruginosa’s* repertoire of secretion systems (type II, type III, type IV, and type VI) and their role in the disease onset. Type III Secretion System (T3SS) effectors contribute, for example, to *P. aeruginosa* pathogenesis in wounds. The *P. aeruginosa* T3SS effector protein Exotoxin T acts as an anti-inflammatory agent by interrupting phosphorylation cascade through tyrosine kinase Abl/PKCδ kinase/inflammasome subtype NLRC4. This impairs NLRC4 inflammasome activation by targeting CrkII, which is required for both Abl transactivation and NLRC4 inflammasome activation [31].

Moreover, human immune cells contain C-type lectins receptors, Dendritic Cell-Specific Intercellular adhesion molecule-3-Grabbing Non-integrin (DC-SIGN), mannose receptor (MR), and Dectin-2, that recognize and bind *P. aeruginosa* biofilm carbohydrates (e.g. Psl and/or Pel). DC-SIGN strongly recognizes *P. aeruginosa* biofilms and planktonic cells while MR and Dectin-2 weakly recognize biofilms. Yet, interference with the endocytic activity of cell-associated DC-SIGN and MR and hindrance of Dectin-2-mediated cellular activation by biofilm carbohydrates, especially those containing a high percent of mannose, can lead to immune evasion [30].

Riquelme et al. unveiled that *P. aeruginosa* escapes immune clearance in infected lungs and persists in the inflamed human airway by redirecting its metabolism to promote biofilm formation and significantly augment synthesis of EPS to the detriment of LPS in the presence of the host macrophages-derived immunometabolite itaconate [32]. EPS shelters *P. aeruginosa* from itaconate-triggered membrane stress and stimulates human myeloid cell metabolic reprogramming, both locally and in circulating monocytes, to trigger even greater itaconate delivery, making the host immune response permissive to chronic infection [32].

Beyond virulence-related factors, such as QS, flagella [33] and formation of biofilm, whose matrix proteome is enriched in proteins involved in oxidation–reduction processes, proteolysis, and transmembrane transport [34], the virulence of *P. aeruginosa* can also be controlled by multiple biological factors. These factors include pyoverdine production (siderophore), *lasR* gene presence, capsule, alginate D, elastase B, exotoxin A and Transcription factors (TFs), with noticeable regulatory roles during pathogenesis [35–37]. For example, RsaL, QscR, RhlR, CdpR, MvfR, PchR, PhoB and LasR were functionally unveiled as master regulators of QS, ExsA was notified as the master regulator of T3SS, and GacA was described as a key regulator of T6SS [37]. Moreover, a recent article detailed the key role played by AlgKX protein complex in alginate production and biofilm attachment in *P. aeruginos*a PAO1 [38].

### Additional factors contributing to *Pseudomonas aeruginosa* survival and infections

*P. aeruginosa* genome encodes two functional DksA (DnaK suppressor protein) paralogs, which confer resistance to oxidative stress. DksA1, containing a zinc-finger motif, is essential for H_2_O_2_ tolerance in both planktonic and biofilm growing cells, and allows the escape of *P. aeruginosa* from macrophages-mediated killing activities via regulation of the genes *katA* and *katE*. DksA2, on its side, is expressed only under zinc starvation and can replace the protective function of DksA1 against oxidative stress [39].

It has also been proven that the acquisition of molybdate through the Type VI secretion system (T6SS), endowed with the ability to secrete an anion-binding protein, confers to *P. aeruginosa* a competitive advantage over the surrounding bacterial species under anaerobic conditions [40]. *P. aeruginosa* also possesses a T6SS toxin (Tse8) that interacts with VgrG1a, component of the VgrG1a-tip complex, for its delivery into target cells where it restricts their ability to synthesize proteins [41].

In the context of co-infecting pathogen communities, the augmented mutation rates (mutators) of *P. aeruginosa* bacteria infecting CF patients are found to be encouraged only in the absence of other species [42]. This illustrates the tremendous advantages that could be endorsed by polymicrobial infections in strategies aiming at *P. aeruginosa* eradication.

Additionally, frequency of resistance emergence in *P. aeruginosa* populations can shift within days based on the nature and duration of antibiotic therapy, since rare mutations not found using culture-based strategies can expand over 5–12 days in riposte to antibiotic changes, while mutations conferring resistance to antibiotics that were not administered decrease and undergo extinction [43]. Another study reported that the higher tolerance of *P. aeruginosa* biofilm cells towards multiple antibiotics, such as gentamicin and colistin, is due to the presence of strains with inactivated *flgE* gene that display cell aggregation, reduced ability to adhere to surfaces, and a faster biofilm growth [44].

### Identification of new potential therapeutic targets against *Pseudomonas aeruginosa* infections

Rapid development of antimicrobial resistance in *P. aeruginosa* as well as the limited efficiency of current antimicrobial treatments on biofilms require the development of alternative antimicrobial strategies to combat *P. aeruginosa* infections. In this perspective, it is essential to identify pathways or mechanisms that are essential for proliferation and/or pathogenicity of this bacterium and that can potentially become new therapeutic targets. We provide here some examples of recent discoveries, which might pave the way to the development of novel antimicrobial strategies towards infections caused by *P. aeruginosa*.

It has been recently confirmed that *P. aeruginosa* possesses a complete denitrification pathway, generating nitric oxide (NO) from NO_2_ supplementation, the supply of endogenous oxygen used in aerobic conditions being directed by NO [45]. Targeting nitrogen sources can be therefore integrated into the strategies designed to eradicate this pathogen.

Even if *P. aeruginosa* exhibits slow growth in CF lung infections [46], reversal to high growth rate in the airways of cystic fibrosis patients enhances antibiotic susceptibility, partially relying on reverse mutations or changes in the transcriptional profile of genes known to be associated with antibiotic resistance [47]. A strategy focusing on reverting the slow growth phenotype of *P. aeruginosa* clinical strains to a high growth rate could therefore be relevant for infections eradication.

Furthermore, to adapt to and thrive within the host system, *P. aeruginosa* modulates the transcription termination stage of its transcription cycle. Indeed, upon induction by small-molecule guanosine tetraphosphate (ppGpp) and in response to DNA damage, the processive antiterminator AlpA positively monitors the expression of the *alpBCDE* genes-encoded programmed cell death pathway by recognizing specific sites on the DNA, and interacts with the β-flap and/or region 1.1 of σ70 RNA polymerase, allowing it to bypass intrinsic termination sites positioned downstream of target promoters. The AlpA also positively monitors the expression of genes in a second putative operon, comprising genes *PA0807*–*PA0829*, to facilitate the survival of *P. aeruginosa* cells in the host [48].

On the other hand, *P. aeruginosa* contains some rhamnolipids (glycolipids molecules) forming micelles that transport both self-produced toxic compounds (pyochelin) and heterologous compounds (e.g. lincosamide antibiotics) for targeting and killing of competing bacterial species (e.g. *Staphylococcus aureus*) during inter-species competition and establishment of the pathogen in its niche [49]. A co-isolated pair of *S. aureus* and *P. aeruginosa* from patients with tracheobronchitis or bronchial colonization revealed that *P. aeruginosa* exoproducts impacted biofilm formation and decreased in vitro growth of its *S. aureus* counterpart, while *S. aureus* did not impair biofilm formation and triggered swarming motility in *P. aeruginosa* [50].

To eradicate infection, the host immune system must sense the presence of the pathogen. In line with this, it has been revealed in the nematode *Caenorhabditis elegans* model of infection that the gut efflux pump multidrug resistance-associated proteins MRP-1, belonging to the C-type family of ATP-binding cassette transporters and showing a high degree of sequence homology to human MRP-1, transports oxidized glutathione, acting as a signalling agent capable of warning *C. elegans* of changes in intestinal homeostasis initiated by the presence of *P. aeruginosa* infection [51].

All these recently described mechanisms involved in *P. aeruginosa* survival and pathogenicity may be exploited in the future as targets for the development of novel antimicrobial agents. Interestingly, targeting mechanisms that are specific to *P. aeruginosa* might allow the development of narrow spectrum antimicrobials, which would specifically inhibit *P. aeruginosa* infection and have no effect on commensal bacteria from the human microbiome.

### Development of diagnostic tools for rapid identification of *Pseudomonas aeruginosa*

In order to efficiently and specifically combat *P. aeruginosa* infections, proper methods for pathogen identification and antimicrobial susceptibility testing (AST) are required. Diagnosis of *P. aeruginosa* infections is usually based on cultures from blood, urine or respiratory samples. AST can then be performed on the isolated strains. Alternatively, rapid tests using real-time quantitative polymerase chain reaction (qPCR) can be used, but cannot differentiate colonization from infection. Recently, based on four novel specific target gene sequences of *P. aeruginosa* identified through pangenome analysis, Wang et al. designed high-specificity and high-sensitivity PCR and qPCR assays for rapid detection of *P. aeruginosa* [52]. qPCR can also provide information about the presence of resistance genes, using a multiplex PCR targeting known resistance genes [1]. However, this does not replace a phenotypic AST, which directly measures the activity of antibiotics on the isolated bacteria. Nevertheless, an important limitation of AST is that it can take up to 48–72 h to identify the suitable antimicrobial treatment.

To respond to the lack of rapid diagnostic protocols for AST, that would allow a timely and rational antibiotic prescription, He et al. designed a specific and rapid reverse assaying protocol for detection and antimicrobial susceptibility testing of *P. aeruginosa* [53]. This method exploits tail fibre protein (TFP)-functionalized magnetic particles for a specific capture of *P. aeruginosa* and a fluorescein isothiocyanate (FITC) labeled magainin II applied as a fluorescent tracer. AST results can be reached within 4 h with this method, avoiding a time-consuming process of bacterial isolation and identification [53].

As failing to detect *P. aeruginosa* early enough is associated with high mortality in immunocompromised patients, the potential biomarkers specific for *P. aeruginosa* infection have been investigated. Xanthine was identified as a potential biomarker and its rapid detection may strongly reduce the time between the onset of symptoms and administration of suitable antimicrobials, which should help avoiding high mortality rates [54]. Moreover, based on the high level and preferential binding of the receptor binding protein GP12, from T4 bacteriophages to the LPS structures on the surface of *P. aeruginosa* cells, this protein has been proposed for *P. aeruginosa* detection in future diagnostic and therapeutic applications [55]. On its side, the Enc34 endolysin from bacteriophage Enc34, containing an N-terminal enzymatically active endolysin domain and a C-terminal transmembrane domain, displays a peptidoglycan-degrading activity towards outer membrane-permeabilized *P. aeruginosa* PAO1 [56]. These two aforementioned proteins can be valuable tools for clinical surveillance and medical-based research. On the other hand, a portable analyser using silica bead-based nucleic acid extraction, and 8-plex real-time reverse transcription loop-mediated isothermal amplification (RT-LAMP) could detect *P. aeruginosa* with high sensitivity in less than 2 h [57].

### Antimicrobial methods to circumvent *Pseudomonas aeruginosa* infection

In the last few years, diverse treatment strategies have been developed in order to circumvent infections caused by *P. aeruginosa* (Table 1). Some antibiotics efficiency towards *P. aeruginosa* infections could be improved by context-specific actions. Indeed, in ventilator-associated pneumonia, combination of cephalosporin and beta-lactamase inhibitor ceftolozane/tazobactam (C/T) exhibited both efficacy and safety in treating extensively drug-resistant *P. aeruginosa* [58]. However, antibiotic resistance against such a combination has been reported when administered at suboptimal steady-state concentrations of 20 mg/L in the susceptible *P. aeruginosa* ST175 isolate [59]. It has also been established that the acquisition of OXA β-lactamases such as OXA-10, and OXA-50, ESBLs GES-1, GES-15, and VEB-1, as well as metallo-β-lactamases (IMP-15, NDM-1, and VIM-2) rendered *P. aeruginosa* isolates resistant to C/T [60].

**Table 1:** Promising methods for eradicating *Pseudomonas aeruginosa* infections stages

Strategies/combinations	Targets	Results	References
Octenidine dihydrochloride-based antiseptic (OCT) and rotating magnetic field (RMF) of two frequencies, 5 and 50 Hz	Biofilms	Biofilm destruction	[61]
Graphene oxide-lignin/silk fibroin/ZnO nanobiocomposite	Biofilms	Prevented biofilm formation	[62]
Combined colistin, AgNPs and decellularized human amniotic membrane (dHAM)	*P. aeruginosa* from burn wounds	Faster wound reduction, presence of considerable fibrosis, complete epithelial reorganization and absence of bacteria on day 21	[63]
Chimeric bacteriocin S5-PmnH		Abolished strain resistance, reduced bacterial numbers, eradicated cytotoxic strain and prevented acute disease	[64]
Anamorphous coatings modified with Cu_2_O nanofibers (coating PC)	Bacterial adhesion	Cytoplasmic outflow and cell membrane destruction, killing effect of Cu^+^ ions	[65]
C16-terpene dilactone (CJ-14445) from *Neofusicoccum luteum*	Bacteria colonies	Antibacterial activity	[66]
Fluorothiazinon	Type III Secretion System (T3SS)	Suppressed the T3SS without affecting bacterial growth	[67]
Synthetic smectite clay minerals and Fe-sulfide microspheres	Bacteria cells	Maintainance of Fe^2^^+^ solubility and reactive oxygen species production, bacteria killing	[68]
Essential oil from *Elsholtzia beddomei* C. B. Clarke ex Hook. f	Bacterial growth	Antibacterial inhibitory effects	[69]
Iodine-loaded polymers I2@NRPOP-1 and I2@NRPOP-2	Bacterial growth	Growth inhibition	[70]
Zinc oxide nanoparticles (ZnO NPs)		Growth inhibition Disruption of cytoplasmic membrane Generation of reactive oxygen species (ROS)	[71]
*Parkia timoriana* (Yongchak/Zawngtah) extract	Bacterial growth	Growth inhibition	[72]
K_3_[Ga(ox)_3_]·3H_2_O and K_4_[Ga2 (ox)_4_ (μ-OH)_2_]·2H_2_O	Bacterial growth	Growth inhibition	[73]
Metallic nanoparticles (MNPs) dip-coating	Bacteria cells	Significant bacterial killing behavior	[74]
Biosynthetic silver nanoparticles (AgNPs) based on *Gmelina arborea* logging residue (GA-AgNPs)	Biofilms and Bacterial growth	Antibacterial, antibiofilm, antioxidant, and wound healing properties, non-toxicity to mammalian cells	[75]
Intraocular implant, MXF-HA, combining hyaluronic acid (HA) and moxifloxacin (MXF) and settled in the eye	Bacterial growth	Growth inhibition	[76]
N-(2-hydroxyphenyl)-2-phenazinamine from marine actinomycete *Nocardiopsis exhalans*	Biofilms	Excellent biofilm inhibitory activity	[77]

#### Methods increasing drug permeation of bacterial cell envelope

As cell envelopes of Gram-negative bacteria act as barriers against exogenous antimicrobial agents, some efforts have been directed toward understanding how small molecules may break through these barriers. A study focused on identifying the permeation potential of compounds towards *P. aeruginosa* and unveiled antibiotics fluoroquinolones and derivatives (prulifloxacin and norfloxacin), linezolid, sulfamethazine, the alkaloid ergotamine and the peptidase inhibitor sitagliptin, as permeators efficiently crossing the epithelial barrier and permeabilizing bacterial membranes [78].

#### Strategies for inhibiting biofilm formation and quorum sensing

Quorum sensing (QS) is an important regulator of virulence factors expression and biofilm formation. Many efforts have been done recently to develop so called “anti-virulence” therapies, by targeting QS signalling.

Indeed, the Dyer Ex Eichler extract (DSE) from the plant *Dioon spinulosum*, was reported to lower biofilm formation, cell surface hydrophobicity, and EPS accumulation of *P. aeruginosa* isolates. It also reduces the relative expression of four QS genes (*lasI*, *lasR*, *rhlI*, *rhlR*) and the biofilm-related gene *ndvB* [79]. On its side, the combination of cinnamaldehyde with gentamicin represses acyl-homoserine lactones production and downregulates the expression of critical QS genes, to substantially diminish pyocyanin, alginate, rhamnolipid, hemolysin, protease, and elastase production, to successfully suppress preformed biofilms and to impair biofilm formation by disrupting EPS production [80].

Other molecules with anti-biofilm and/or anti-QS features have also been described. For example, the newly synthesized molecules, pyridine derivative 3, amino benzenesulfonamide derivative 2, furothiazole derivative 4, and thienothiazole derivative 5, exhibit potent biofilm inhibition activity against *P. aeruginosa* ATCC 27853. However, the pyridine derivative 3 has a similar efficiency against *P. aeruginosa* ATCC 27853 to that of reference drugs, ampicillin, and ciprofloxacin [81]. Compounds (e.g. Aurine tricarboxylic acid) that may impair EPS protein-eDNA interaction can also be specifically effective in eradicating biofilms generated by RSCVs [12]. Furthermore, N-Aryl Malonamides (NAMs) were recently described as potent compounds inhibiting the QS transcriptional regulator MvfR and thus controlling virulence [82].

*P. aeruginosa* can escape antimicrobials threat by trapping the antibiotics in the biofilm extracellular matrix. To overcome this, dextran-based single-chain polymer nanoparticles (SCPNs) coupled with DNase I and associated with tobramycin have been applied to disperse the biofilm matrix. This enhances the activity of tobramycin and DNase I on *P. aeruginosa* biofilms by neutralizing the ionic interactions that retain this antibiotic in the biofilm periphery [83].

#### Natural molecules reducing *Pseudomonas aeruginosa* growth

**Fig. 1:**
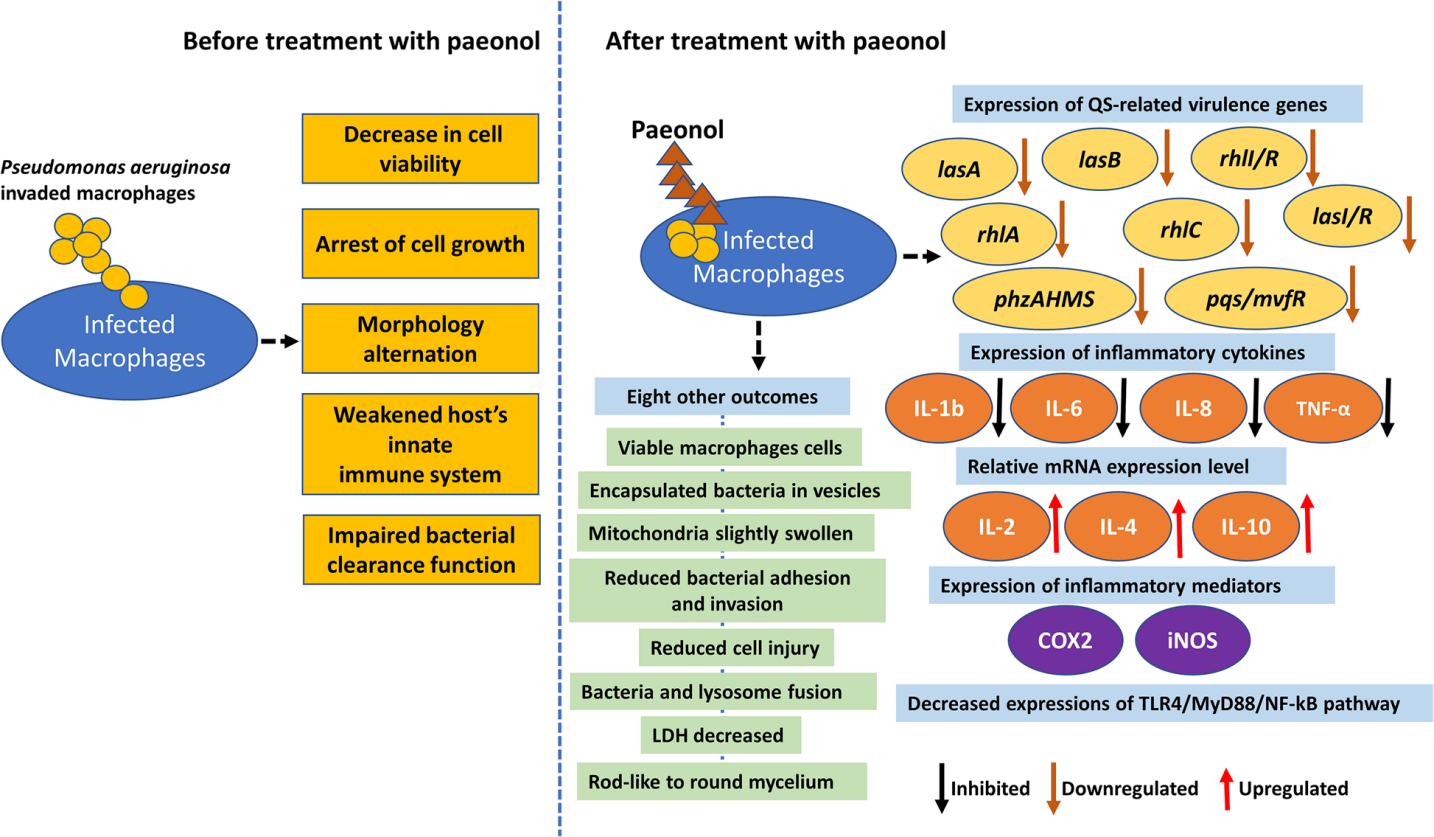
Paeonol effects on *Pseudomonas aeruginosa* infected macrophages. Based on the work of Tang H et al. (2022) [92]. Pathogen bacteria and their genes are in yellow, macrophage is in blue and the components of immune system in other colours

Although synthetic compounds can drastically reduce *P. aeruginosa* growth (e.g. phosphate-based coacervates containing metallic antibacterial ions Ag^+^ [84]), natural molecules can be highly efficient, safe, cost-effective and environment friendly. The natural compounds 6-gingerol and curcumin have been unveiled to inhibit QS activation and production of biofilm, EPS, pyocyanin, and rhamnolipid along with enhancing the susceptibility of *P. aeruginosa* AM26 to antibiotics such as ciprofloxacin and ceftazidime hydrate [85]. On the other hand, pure compounds from plants have been reported to exert antimicrobial activities against clinical isolates of *P. aeruginosa*. Indeed, molecules extracted from the leaf of the plant *Andrographis paniculata* (Burm f.) Nees, andrographolide, 14-deoxyandrographolide, 14-deoxy-12-hydroxyandrographolide, and neoandrographolide, exert QS quenching activity, inhibiting biofilm formation, protease production, and swarming motility of the clinical isolates of metallo-β-lactamase producing *P. aeruginosa* PA22 and PA24 [86]. Paeonol, also known as 2′-hydroxy-4′-methoxyacetophenone, is a phenolic acid molecule isolated from the root bark of traditional Chinese medicinal herbs, such as *Arisaema erubescens* [87]. It has been demonstrated to exert a wide range of activities, spanning from immune regulatory activity [88], anti-inflammatory [89], to antibacterial effect [90, 91]. Paeonol efficiently reduces infection of macrophages by *P. aeruginosa*, as described recently [92]. Furthermore, it has the potential to drastically impede the expression of QS-regulated gene *rhlI/R* and *pqsA/R* [93]. Detailed effects of paeonol on infected macrophages are described in Fig. 1.

**Fig. 2:**
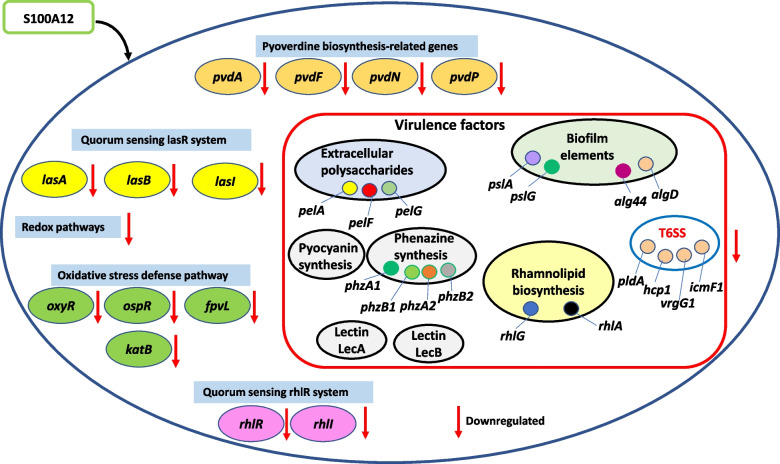
Genes or pathways affected in *Pseudomonas aeruginosa* PAO1 in response to S100A12 treatment. The exact mechanism of action of S100A12 is not known, but it causes a broad transcriptional response. The genes of pyoverdine biosynthesis are downregulated in *P. aeruginosa* PAO1 in response to S100A12 treatment. *pvdA*, encoding the enzyme L -ornithine N5-oxygenase, induces the initial step in the pyoverdine synthesis pathway, *pvdP* turns ferribactin into fluorescent pyoverdine, *pvdF* and *pvdN* are important genes in pyoverdine biosynthesis. Quorum sensing *lasR* system related genes are impeded. Metabolic and redox pathways along with virulence factors as well as transporter and membrane proteins are also impeded. Reduced expression of rhamnolipid biosynthesis related genes *rhlG,* encoding an NADPH dependent ketoacyl reductase and *rhlA, rhlG,* as well as QS *rhlR* system *rhlR, rhll-* are reduced. Extracellular polysaccharides genes *pelA*, *pelF,* and *pelG* as well as biofilm genes *pslA*, *pslG*, *algD*, and *alg44* are downregulated. Oxidative stress defense pathway genes, *oxyR*, *ospR*, *fpvL* and *katB* are downregulated. Expression of genes implicated in phenazine synthesis pathway, *phzA1*, *phzB1*, *phzA2*, and *phzB2* are drastically reduced. Expression of genes related to the structural component of type 6 secretion system (T6SS); *pldA*, *icmF1*, *hcp1*, and *vrgG1,* are also diminished. This figure is based on Mishra P et al. (2022) [98] publication

Silver nanoparticles produced using extracts from the berries of the plant *Ligustrum vulgare* have also been proven to exhibit antibacterial ability against *P. aeruginosa* [94]. Moreover, naturally derived hydroquinone has been found to rapidly disrupt the cell membrane, increase permeability and provoke leakage of intracellular potassium ions in *P. aeruginosa* MTCC 741 [95]. Furthermore, the marine organism brittle star *Ophiocoma dentata* crude extract and derived sesquiterpenoids molecules 8,11-epoxy-9(15)-himachaladiene-4-ol(O8-ophiocomane) and 11-epoxy-9(15)-himachaladiene-4-ol (O7-ophiocomane), exert noticeable antimicrobial effects against *P. aeruginosa* [96]. It is worth noting that human body naturally produces antimicrobial peptides that can combat infections driven by bacterial pathogens. For example, the antimicrobial peptides S100A12 (calgranulin C) is significantly expressed in immune cells like neutrophils and macrophages in addition to corneal tissues of patients with *Pseudomonas* keratitis [97, 98]. Its roles on *P. aeruginosa* growth, biofilm formation, pyoverdine secretion and type VI secretion system have been depicted in Fig. 2.

#### Additional methods for restricting *Pseudomonas aeruginosa* infections

Other methods for controlling *P. aeruginosa* infections implicate the use of bacteriophages whose host specificity and mode of infectivity depend on the interactions between the viral proteins and the surface of the host bacteria. Nodstrom et al. proposed bacteriophage therapy as a promising alternative to eradicate biofilms formed by genetically diverse *P. aeruginosa* clinical strains isolated from cystic fibrosis patients [99]. The lytic *Pseudomonas* phage LUZ19 targets QS of *P. aeruginosa* via a QS targeting protein, Qst. Qst interacts with PqsD, a key enzyme of the quinolone biosynthesis pathway. This causes a decrease in levels of *Pseudomonas* quinolone, allowing efficient LUZ19 infection [100]. Another mechanism, which might inspire the development of innovative antimicrobial strategies, is involved in *Pseudomonas* bacteriophage LUZ24 activity. This bacteriophage produces a peptide, Igy, that interacts with the gyrase GyrB, impairing its activity and blocking DNA replication even in fluoroquinolone-resistant *P. aeruginosa* isolates [101].

Another study describes the role of PP-007, a polyethylene-glycol-modified (PEGylated) bovine hemoglobin-based CO carrier, in priming monocytes/cystic fibrosis-affected macrophages to express high levels of heme oxygenase-1 to stimulate the resolution of neutrophilic pulmonary inflammation without compromising the clearance of *P. aeruginosa* [102].

Considering that antibiotic resistance of pathogens can lead to high mortality of the host, two main strategies have been envisioned to combat multiresistant bacteria: the restoration of efficiency of antibiotics that have been rendered ineffective due to the increasing rate of antibiotic resistance and the modification of existing antimicrobial agents to design new compounds with superior efficacy. For example, Hochvaldová et al. improved and restored the antibacterial activity of antibiotics (gentamicin, ceftazidime, ciprofloxacin, and colistin) by applying them together with a synthesized cyanographene/Ag nanohybrid [103]. They reported the antibacterial efficiency of this combined treatment against *P. aeruginosa* strains and determined that both antibiotic's modes of action and mechanisms of bacterial resistance strongly impacted the combined treatment’s efficacy. When the FDA-approved antispasmodic drug, Otilonium bromide (IUPAC: N,Ndiethyl-N-methyl-2-[(4-benzoyl)oxy]ethanaminium) was simultaneously applied with the last-line antibiotic colistin on *P. aeruginosa* strains, it restored the antimicrobial effects of colistin. The two compounds act synergistically to permeabilize bacterial cell membranes, dissipate proton motive force, inactivate efflux pumps, and induce membrane damages, cytosol leakage and cell death [104]. On their side, in order to improve efficiency of polymyxin B (PMB) and colistin and decrease their toxicity, Roberts et al. modified multiple non-conserved positions within the polymyxin scaffold to design the synthetic lipopeptide F365 (QPX9003) displaying a wider therapeutic window, reduced nephrotoxicity and toxicity, improved pharmacokinetics properties, and efficacy against lung infections caused by top-priority multi-drug resistant pathogens including *P. aeruginosa* [105]. Peptides are becoming increasingly important for many therapeutic areas [106], due to their capacity to permeate tissues and membranes and the low rate of resistance emergence towards them. The de novo*-*engineered cationic peptide antibiotic E35, for example, irreversibly damages cell membranes and kills the extensively-resistant isolate PA239 [107].

Polytherapy with an antibiotic and a lyotropic liquid-crystalline lipid-based nanoparticle carrier has been successfully applied to kill *P. aeruginosa* and the mechanism of action of the two members of this therapy has been described. Indeed, combined treatment of PMB and cubosomes intensifies *P. aeruginosa* bacterial killing as PMB initiates the disorganization of the outer membrane of the target bacteria, and thereafter, an influx of cubosomes further accelerates membrane disruption through a lipid exchange process [108].

Many potential antimicrobial agents that were developed in vitro did not reach clinical application, since their in vivo activity was strongly diminished compared to their in vitro efficacy. To understand the reduced efficacy of antibiotics against *P. aeruginosa* in vivo, a study focused on the impaired diffusion of the antibiotic colistin across an artificial sputum matrix/medium and quantified its antimicrobial activity against *P. aeruginosa* NH57388A biofilms. Stokniene et al. revealed that the binding of colistin to mucin-rich AS medium substantially diminished its diffusion rate and reduced its effectiveness. On the other hand, the addition of the low molecular weight alginate oligosaccharide OligoG CF-5/20, derived from the stem of brown algae *Laminaria hyperborean*, enhanced colistin diffusion in mucin-rich AS medium, enhanced mucus penetration by colistin, and significantly intensified colistin antimicrobial activity against mucoid *P. aeruginosa* biofilms [109]. Of note, colistin is known to bind to mucin in CF sputum or on the airway epithelium [110], and its bactericidal activity is mediated by the electrostatic interplay between its cationic amino groups and LPS anionic phosphate groups on the outer membrane of the targeted Gram-negative bacteria. O’Brien et al. further highlighted that the growth in a polymicrobial environment shields the target microorganism from the effect(s) of antimicrobial agents [111]. The same authors identified a single nucleotide polymorphism as well as indels in genes encoding LPS biosynthesis and/or pilus biogenesis in colistin-resistant *P. aeruginosa* isolates and also reported that loss-of-function mutations (e.g. frameshifts and nonsense mutations) in the genes implicated in LPS biosynthesis (eg. *wzy* gene) contributed to the resistance mechanism towards colistin.

While the search for efficient antibiotics continues, some works focused on compounds that do not induce antimicrobial resistance selection and are deprived of significant cytotoxicity on mammalian cells. For example, vitamin C administration is found to display remarkable antibacterial and anti-biofilm features against *P. aeruginosa* [112]. Furthermore, the combination of epsilon-poly-L-lysine and antibiotics ampicillin, gentamicin, tetracycline, or methicillin, inhibit formation of *P. aeruginosa* biofilm and improve preformed biofilm disruption in vitro with no significant cytotoxicity in fibroblasts [113]. On the other hand, microwave plasma-activated water was shown to exert bactericidal activities against *P. aeruginosa* with no harmful effects on normal skin cells [114]. This could therefore be used as an efficient and safe skin disinfectant. Antimicrobial hybrid peptide Lf-KR exhibits increased permeabilization and depolarization of microbial membranes, the ability to substantially impair expression and production of pro-inflammatory cytokines (nitric oxide and tumor necrosis factor‐α) in LPS-stimulated mouse macrophages and a powerful suppressing effect on preformed multidrug-resistant *P. aeruginosa* biofilms [115].

A method increasing the sensitivity to various antibiotics and that can be used as an alternative to conventional antibiotics has also been investigated. Indeed, a sensitizing approach employing cell-penetrating peptides conjugated with peptide nucleic acid and targeting *bamB* (encoding an outer membrane lipoprotein) and *oprM*, a *tolC* homolog encoding an outer membrane efflux protein, has been reported to enhance the uptake of vancomycin, erythromycin and carbenicillin by *P. aeruginosa* [116]. This finding confirms that manipulating outer membrane transport in *P. aeruginosa* can enhance its susceptibility to antibiotics.

### Vaccine: a tool to prevent infections by antibiotic-resistant *Pseudomonas aeruginosa*

Development of vaccines can be an alternative to prevent infections and thus avoid excessive use of antibiotics leading to antibiotic resistance. Vaccines targeting T3SS translocons (V-antigen PcrV), exoenzymes (ExoS, ExoU), fimbrial components, flagella, core LPS [117], outer membrane vesicles components [118], recombinant lipoprotein I (OprI, [119]), and alginate deriving from a synthase-dependent exopolysaccharide secretion system [120], have received great attention as they can provide protection against infection and disease onset [121–123]. For example, a trivalent vaccine based on outer membrane proteins (OprF and OprI) and T3SS translocon protein (PopB) of *P. aeruginosa*, and formulated with or without Granulocyte–macrophage colony-stimulating factor (GM-CSF) as an adjuvant, is found to stimulate Th1 and Th2 responses, to increase the secretion of immunoglobulin A (IgA), and to induce proper level of IgG (G1, G2a, G2b) against *P. aeruginosa* in the burned rat models [124].

Strategies focusing on notorious virulence factors that aid in the evasion of the host immune response represented the predominant steps in vaccine development towards *P. aeruginosa*. In this regard, a report highlighted that LPS and Oligopolysaccharides (OPS) antigens conjugated with Poly Lactic-co-Glycolic Acid (PLGA) nanoparticles have the potential to be employed as nano-vaccines stimulating cellular and humoral immune systems against *P. aeruginosa* infections. Indeed, LPS-PLGA and OPS-PLGA conjugates triggered immunoglobulin M (IgM), IgA, immunoglobulin G (IgG), IgG1, IgG2b, IgG2a and IgG3 antibodies production and facilitated an effective immunity against *P. aeruginosa* in a conjugate dependent manner. In fact, mice vaccinated with LPS-PLGA conjugates produced higher levels of anti-LPS-PLGA antibodies and were more efficiently protected towards *P. aeruginosa* infections than mice vaccinated with OPS-PLGA conjugates [125]. By loading cytosolic antigens derived from bacterial lysates of *P. aeruginosa* PAO1 strain onto mesoporous silica nanospheres decorated with membrane antigens derived from double-layered membrane vesicles of the same bacterial strain (PAO1), an efficient nano-vaccine has been created. This nano-vaccine was reported to trigger humoral as well as cellular immune responses that significantly prevented infections in mice by the drug-resistant *P. aeruginosa* PAO1 and PA-XN-1 strains [126]. As alginate of *P. aeruginosa* mediates pathogenesis in host cells, one main target of efforts is to design therapeutic vaccines. Immunity against *P. aeruginosa* in mice has also been induced by using mannuronic acid tetrasaccharide, as antigen epitope for vaccine development [127].

Furthermore, hybrid proteins composed of the full-length V-antigen (PcrV) and C-terminal domain exoenzyme S (ExoS) from *P. aeruginosa*, coupled with adjuvants alum and monophosphoryl Lipid A, have been used as vaccine candidates to protect mice against urinary tract infections caused by *P. aeruginosa* strain PAO1. This treatment enhanced the levels of humoral (IgG1, serum anti-protein IgA, mucosal IgG), and IL-17 production in the vaccinated mice [128]. Noteworthy, whole-cell vaccine inactivated by X-ray irradiation, containing nucleic acids and 8-hydroxyguanosine, was reported to trigger a humoral response in dentritic cell (DCs) that prevented infection by *P. aeruginosa* PAO1 and multidrug-resistant clinical isolate W9 in mice model of pneumonia. In addition, this treatment induced cGAS-STING pathway triggering (innate immune response), modulation of Toll-like receptors, apoptosis, pyroptosis, CD8^+^ T-cell proliferation, Th1 and Th2 cytokine responses, and reduced levels of inflammatory factors (IL-6, TNF-α and IL-8) in DC [129]. Another report confirmed that vaccination of mice with *P. aeruginosa’s* outer membrane vesicles (PA-OMVs) conjugated with the diphtheria toxoid (DT) formulated with alum adjuvant, namely PA-OMVs-DT + adj conjugated, resulted in a lower bacterial load, drastic decrease of inflammatory cell infiltration with less tissue damage and conferred an efficient protection against *P. aeruginosa* in the mice burn model [130]. Of notes, OMVs are particularly attractive as a vaccine platform due to their non-replicating nature, ability to accumulate in lymph nodes, their natural and innate composition of pathogen-associated molecular patterns, and ability to be produced in large quantities by bacterial fermentation [131, 132].

Additionally, Rahbar et al. designed a triple-target antigen that could stimulate simultaneous protective and neutralizing antibodies against COVID-19 responsible virus, respiratory syndrome coronavirus 2 (SARS-CoV-2), and associated bacterial pathogens causing nosocomial infections, such as *A. baumannii* and *P. aeruginosa* [133]. The designed antigen was made by combining epitopes originating from *A. baumannii* outer membrane protein A (*Ab*OmpA), OprF (*P. aeruginosa* outer membrane protein F), and foreign multi-epitopes (SARS-CoV-2 Spike glycoprotein).

Vaccines designed to antagonize the virulence of drug-resistant *P. aeruginosa* aimed at preventing chronic infection and pathogenesis exacerbation, and conferring post-exposure immunization. Although the biosecurity of these vaccines has not been fully elucidated for human, the available vaccines represent a promising approach for the prevention of *P. aeruginosa* infections. For example, purified recombinant fragment of the OprL (reOprL) of *P. aeruginosa* has been employed as vaccine to elicit a strong pulmonary response of specific effector T cells deriving from naive T cells, namely Th17 cells, leading to serotype-independent protection against acute lung infection of mice by *P. aeruginosa* [134]. In the meanwhile, the intranasal injection of the primate-based AdC7 vector AdC7OprF.RGD, expressing outer membrane protein F (OprF) of *P. aeruginosa* (AdC7OprF) and exhibiting an integrin-binding arginine–glycine–aspartic acid (RGD) sequence, immunized a mouse model of cystic fibrosis against established *P. aeruginosa* respiratory infection and eliminated *P. aeruginosa* from the lungs [135]. A trivalent combination DNA vaccine based on *oprL*, *oprF* and *flgE* genes of *P. aeruginosa* has also been unveiled to trigger a robust humoral immune response (higher levels of IFN-γ, IL-2, and IL-4), lymphocyte proliferation and protective efficacy in immunised chickens [136]. For reaching a protective efficacy in vaccinated chickens, divalent combination DNA vaccine (pOPRL and pOPRF) had to be employed at an optimal immunization dose (100 and 200 µg doses) to control infection driven by *P. aeruginosa* [137]. This finding illustrates vaccines limitations in achieving protective immunogenicity.

Vaccines can induce a protective response against a given pathogen via either active or passive immunization. It has been shown that vaccination prevents infections by other pathogens via the release of cross-reactive antibodies that bind closely-related antigens in other organisms. For example, mice vaccinated with recombinantly produced *Bordetella pertussis* OmpA protein are protected against *P. aeruginosa* PAO1 pneumonia and sepsis [138]. The same report demonstrated that *B. pertussis* whole cell vaccine (*Bp-*WCV) mitigated *P. aeruginosa* PAO1 bacterial burden in the airways of mice, triggered anti-*P. aeruginosa* IgG production, and antibodies generated against *B. pertussis* also recognized clinical *P. aeruginosa* strains [138]. This finding also suggests that whole cell vaccine targeting a given pathogen can train the immune system to develop immunological response upon exposure to a different pathogen.

The development of preventive therapies, including the design of novel vaccines, has been reported to require specific carbohydrates [139]. A recently developed *P. aeruginosa* glycoconjugate vaccine, containing bacterial core lipopolysaccharide tetrasaccharide Hep2Kdo2 attached via a chain linker to a diphtheria toxin mutant carrier protein, binds the cell-surface sugars of *Pseudomonas aeruginosa* and facilitates bacterial killing [117]. Glycoconjugate vaccines are highly promising for the clearance of many important human pathogens due to their ability to trigger both T-cell-dependent and T-cell-independent immune responses [140]. It is worth noting that carrier proteins utilization can be hindered by some limitations: (i) a restricted number of existing carrier proteins is applicable in licensed conjugate vaccines (e.g., DT, CRM197, Outer Membrane Protein Complex), and (ii) repeated exposure to the same carrier may provoke diverse immune interferences including carrier-specific enhancement of T cell help, carrier-induced epitope suppression and bystander interference [141, 142]. Fortunately, alternative carrier proteins candidates including recombinant non-toxic form of *P. aeruginosa* exotoxin A (rEPA) and recombinant proteins containing strings of universal CD4^+^ T-cell epitopes have been developed and can overcome those limitations [143].

Protein engineering of the natural polyhydroxyalkanoate (PHA) production system of *P. aeruginosa* PAO1 has been proven extremely relevant to display selected antigens that can be employed as vaccines candidates against *P. aeruginosa* infections. Indeed, the deletion of key genes coding for the synthesis of PHA inclusions, alginate, and pel polysaccharide, allowed to improve production of PHA beads coated with surface epitopes of vaccine candidates outer membrane proteins AlgE, OprF, and OprI. These PHA beads coated with OprI/F-AlgE fusion antigen elicited both Th1 type immune response illustrated by the production of IFN-γ and IgG2c isotype antibodies and opsonophagocytic killing mediated by sera antibodies in the vaccinated mice [144].

### Monoclonal antibodies neutralizing the activity of *P. aeruginosa* strains

Aside from vaccines and antibiotics, monoclonal antibodies (mAbs) have been deployed to control the spread of *P. aeruginosa* and lower infection severity. mAbs are viewed as a treatment option for high-risk individuals for whom vaccination is not an option and passive administration of mAbs could have a major effect on *P. aeruginosa* pathogenesis by conferring immediate protection, thus complementing the effect of prophylactic vaccines. For example, to overcome canonical antimicrobial resistance of biofilm-resident bacteria, monoclonal antibodies that can release *P. aeruginosa* and its common co-pathogens from the protective biofilm for subsequent killing by antibiotics have been developed [145]. Indeed, monoclonal antibodies directed against DNABII protein epitopes or targeting type IV pilus from the respiratory tract pathogen *Haemophilus influenzae* significantly impaired biofilms of *P. aeruginosa* and related respiratory tract pathogens (*Burkholderia cenocepacia*, *Staphylococcus aureus*, *Streptococcus pneumoniae* or *Moraxella catarrhalis* [145]). Among the mAbs candidates that have been examined, chicken egg yolk immunoglobulins IgY antibodies, have drawn a special interest in passive immunization due to a wide range of features encompassing the absence of immunological cross-reactivity with mammalian IgG and the complement system, high levels of antigen-specific production yield without disease resistance, and ability to facilitate immunization methods without stress in human [146, 147]. More specifically, IgY raised against the T3SS translocating protein, recombinant PcrV from *P. aeruginosa* PAO1 strain, allowed to generate Anti-PcrV IgY for immunization of hen. This Anti-PcrV IgY augmented opsonophagocytic killing and repressed bacterial invasion in *P. aeruginosa* murine acute pneumonia and burn wound models [148]. A synergistic action between anti-*P. aeruginosa* IgY and beta-lactams (ceftazidime, imipenem, and meropenem) has been recently unveiled and raises the possibility to combine antibodies and antibiotics for treatment of infections by multi-drug resistant *P. aeruginosa* [149].

The mAbs can be coupled with antibiotics to achieve superior therapeutic efficacy for severe *P. aeruginosa* pneumonia. In this regard, the DNA-delivered monoclonal antibodies (DMAbs) produced in vivo by skeletal muscles and containing potent human IgG clones as well as non-natural bispecific IgG1 candidates targeting *P. aeruginosa* strain 6077 have been proven to protect mice against lethal pneumonia caused by aggressive *P. aeruginosa* strains [150]. DMAbs reduced bacterial colonization of organs (spleen, kidneys), prevented pulmonary oedema, acted synergistically with a commonly used carbapenem family antibiotic (meropenem), was temperature stable and is proposed to be suitable for treating high-risk patients with chronic diseases, and pathogens that are refractory to many broad-spectrum antibiotic regimens [150].

Although therapeutic monoclonal antibodies are reported as promising methods to restrict *P. aeruginosa* pathogenesis, they can display some limitations. For example, the bivalent, bispecific human immunoglobulin G1 kappa monoclonal antibody MEDI3902 (gremubamab) failed to mitigate *P. aeruginosa* nosocomial pneumonia incidence in *P. aeruginosa*-colonised mechanically ventilated subjects [151]. Moreover, passive immunization by monoclonal antibodies such as IgY raised against chimeric protein pilQ-pilA-DSL region in *P. aeruginosa* also failed to protect rabbits against sepsis [152]. Immunogenicity and protective efficacy of IgY antibodies can also be dose-dependent and non-type specific. In fact, IgY antibodies raised against recombinant type A flagellins of *P. aeruginosa* did not protect mice in burn wound model, but conferred full protection against *P. aeruginosa* PAK and PAO1 in acute pneumonia challenge [153]. Therefore, passive immunization by polyclonal antibodies or direct administration of higher dose of mAbs could be envisioned as a safer alternative against bacterial infections.

## Conclusions

*P. aeruginosa* deploys a range of virulence-associated and adaptive mechanisms to subvert the host system during infection. New methods for developing treatments that can offer substantial benefits for patients with serious, unmet medical needs have been covered in this review. Efficient strategies to improve existing antimicrobial agents as well as the discovery of novel molecules with less toxicity have been reported. More viable approaches that may selectively kill bacteria upon contact yet remain nontoxic to mammalian cells or treatment focusing on how the tissue responds to biofilm proliferation, and not merely how effective the treatment is in eradicating the virulence-associated factors merits critical investigation. Natural compounds are of particular interest, since they are a promising source of antimicrobial agents that may allow to improve the clinical management of *P. aeruginosa* infections. Efforts have now to be made in order to bring the recently developed antimicrobial strategies to clinical application, in order to circumvent infections caused by MDR *P. aeruginosa*.

## Data Availability

Not applicable.

## Abbreviations

AST: Antimicrobial susceptibility testing
CF: Cystic fibrosis
C/T: Ceftolozane/tazobactam
DSL: C-terminal disulphide loop
DC-SIGN: Dendritic cell-specific intercellular adhesion molecule-3-grabbing non-integrin
EPS: Exopolysaccharide
LPS: Lipopolysaccharide
MDR: Multidrug resistant
MR: Mannose receptor
NO: Nitric oxide
PMB: Polymyxin B
qPCR: Quantitative polymerase chain reaction
QS: Quorum sensing
QSAI: Quorum sensing autoinducer
RSCVs: Rugose small colony variants
T3SS: Type 3 secretion system
T6SS: Type 6 secretion system
UTI: Urinary tract infection

## References

[1] Reynolds D, Kollef M (2021). The epidemiology and pathogenesis and treatment of Pseudomonas aeruginosa infections: an update. Drugs.

[2] Mulcahy LR, Isabella VM, Lewis K (2014). Pseudomonas aeruginosa biofilms in disease. Microb Ecol.

[3] Kapel N, Caballero JD, MacLean RC (2022). Localized pmrB hypermutation drives the evolution of colistin heteroresistance. Cell Rep..

[4] Krishnamoorthy G, Leus IV, Weeks JW, Wolloscheck D, Rybenkov VV, Zgurskaya HI (2017). Synergy between active efflux and outer membrane diffusion defines rules of antibiotic permeation into Gram-negative bacteria. mBio.

[5] Strateva T, Yordanov D (2009). Pseudomonas aeruginosa - a phenomenon of bacterial resistance. J Med Microbiol.

[6] Lambert PA (2002). Mechanisms of antibiotic resistance in Pseudomonas aeruginosa. J R Soc Med.

[7] Kumari N, Kumar M, Katiyar A, Kumar A, Priya P, Kumar B (2022). Genome-wide identification of carbapenem-resistant Gram-negative bacterial (CR-GNB) isolates retrieved from hospitalized patients in Bihar, India. Sci Rep.

[8] Saki M, Farajzadeh Sheikh A, Seyed-Mohammadi S, AsarehZadegan Dezfuli A, Shahin M, Tabasi M (2022). Occurrence of plasmid-mediated quinolone resistance genes in Pseudomonas aeruginosa strains isolated from clinical specimens in southwest Iran: a multicentral study. Sci Rep.

[9] Asokan GV, Ramadhan T, Ahmed E, Sanad H (2019). WHO global priority pathogens list: a bibliometric analysis of Medline-PubMed for knowledge mobilization to infection prevention and control practices in Bahrain. Oman Med J.

[10] Tuon FF, Dantas LR, Suss PH, Tasca Ribeiro VS. Pathogenesis of the Pseudomonas aeruginosa biofilm: a review. Pathogens (Basel, Switzerland). 2022;11(3). 10.3390/pathogens11030300. 10.3390/pathogens11030300 PMC8950561 35335624

[11] Hall CW, Mah T-F (2017). Molecular mechanisms of biofilm-based antibiotic resistance and tolerance in pathogenic bacteria. FEMS Microbiol Rev.

[12] Deng B, Ghatak S, Sarkar S, Singh K, Das Ghatak P, Mathew-Steiner SS, et al. Novel bacterial diversity and fragmented eDNA identified in hyperbiofilm-forming *Pseudomonas aeruginosa* rugose small colony variant. iScience. 2020;23(2). 10.1016/j.isci.2020.100827. 10.1016/j.isci.2020.100827 PMC6997594 32058950

[13] Taabodi M, May EB, Bryant RB, Saporito LS, Skeen OK, Hashem FM, et al. *Aeromonas hydrophila, Bacillus thuringiensis, Escherichia coli and Pseudomonas aeruginosa* utilization of Ammonium-N, Nitrate-N and Urea-N in culture. Heliyon. 2020;6(4). 10.1016/j.heliyon.2020.e03711. 10.1016/j.heliyon.2020.e03711 PMC7163070 32322713

[14] Gupta KK, Devi D (2020). Characteristics investigation on biofilm formation and biodegradation activities of Pseudomonas aeruginosa strain ISJ14 colonizing low density polyethylene (LDPE) surface. Heliyon.

[15] Rilda Y, Damara D, Putri YE, Refinel R, Agustien A, Pardi H (2020). Pseudomonas aeruginosa antibacterial textile cotton fiber construction based on ZnO–TiO2 nanorods template. Heliyon.

[16] Lau GW, Hassett DJ, Britigan BE (2005). Modulation of lung epithelial functions by *Pseudomonas aeruginosa*. Trends Microbiol.

[17] Gajdos L, Blakeley MP, Haertlein M, Forsyth VT, Devos JM, Imberty A (2022). Neutron crystallography reveals mechanisms used by Pseudomonas aeruginosa for host-cell binding. Nat Commun.

[18] Badaoui M, Zoso A, Idris T, Bacchetta M, Simonin J, Lemeille S, et al. Vav3 Mediates *Pseudomonas aeruginosa* adhesion to the cystic fibrosis airway epithelium. Cell Rep. 2020;32(1). 10.1016/j.celrep.2020.107842. 10.1016/j.celrep.2020.107842 32640241

[19] Jeske A, Arce-Rodriguez A, Thöming JG, Tomasch J, Häussler S (2022). Evolution of biofilm-adapted gene expression profiles in lasR-deficient clinical Pseudomonas aeruginosa isolates. NPJ Biofilms Microbiomes.

[20] Davies DG, Parsek MR, Pearson JP, Iglewski BH, Costerton JW, Greenberg EP (1998). The involvement of cell-to-cell signals in the development of a bacterial biofilm. Science.

[21] Darch SE, Simoska O, Fitzpatrick M, Barraza JP, Stevenson KJ, Bonnecaze RT (2018). Spatial determinants of quorum signaling in a *Pseudomonas aeruginosa* infection model. Proc Natl Acad Sci U S A.

[22] Kumar L, Patel SKS, Kharga K, Kumar R, Kumar P, Pandohee J (2022). Molecular mechanisms and applications of N-Acyl homoserine lactone-mediated quorum sensing in bacteria. Molecules.

[23] Trouillon J, Han K, Attrée I, Lory S (2022). The core and accessory Hfq interactomes across Pseudomonas aeruginosa lineages. Nat Commun.

[24] Ma LZ, Wang D, Liu Y, Zhang Z, Wozniak DJ. Regulation of biofilm exopolysaccharide biosynthesis and degradation in Pseudomonas aeruginosa. 2022;76(1):null. 10.1146/annurev-micro-041320-111355. 10.1146/annurev-micro-041320-111355 35655342

[25] Stoner SN, Baty JJ, Scoffield JA (2022). Pseudomonas aeruginosa polysaccharide Psl supports airway microbial community development. ISME J.

[26] Kaleta MF, Petrova OE, Zampaloni C, Garcia-Alcalde F, Parker M, Sauer K (2022). A previously uncharacterized gene, PA2146, contributes to biofilm formation and drug tolerance across the ɣ-Proteobacteria. NPJ Biofilms Microbiomes.

[27] Bjarnsholt T, Jensen P, Fiandaca MJ, Pedersen J, Hansen CR, Andersen CB (2009). Pseudomonas aeruginosa biofilms in the respiratory tract of cystic fibrosis patients. Pediatr Pulmonol.

[28] Hills OJ, Yong CW, Scott AJ, Devine DA, Smith J, Chappell HF (2022). Atomic-scale interactions between quorum sensing autoinducer molecules and the mucoid P. aeruginosa exopolysaccharide matrix. Sci Rep.

[29] Tahmasebi H, Dehbashi S, Nasaj M, Arabestani MR (2022). Molecular epidemiology and collaboration of siderophore-based iron acquisition with surface adhesion in hypervirulent Pseudomonas aeruginosa isolates from wound infections. Sci Rep.

[30] Singh S, Almuhanna Y, Alshahrani MY, Lowman DW, Rice PJ, Gell C (2021). Carbohydrates from Pseudomonas aeruginosa biofilms interact with immune C-type lectins and interfere with their receptor function. NPJ Biofilms Microbiomes.

[31] Mohamed MF, Gupta K, Goldufsky JW, Roy R, Callaghan LT, Wetzel DM (2022). CrkII/Abl phosphorylation cascade is critical for NLRC4 inflammasome activity and is blocked by Pseudomonas aeruginosa ExoT. Nat Commun.

[32] Riquelme SA, Liimatta K, Wong Fok Lung  T, Fields B, Ahn D, Chen D (2020). Pseudomonas aeruginosa utilizes host-derived itaconate to redirect its metabolism to promote biofilm formation. Cell Metab.

[33] Campodónico VL, Llosa NJ, Grout M, Döring G, Maira-Litrán T, Pier GB. Evaluation of flagella and flagellin of *Pseudomonas aeruginosa* as vaccines. 2010;78(2):746-55. 10.1128/IAI.00806-09. 10.1128/IAI.00806-09 PMC2812208 19995892

[34] Egorova DA, Solovyev AI, Polyakov NB, Danilova KV, Scherbakova AA, Kravtsov IN (2022). Biofilm matrix proteome of clinical strain of P. aeruginosa isolated from bronchoalveolar lavage of patient in intensive care unit. Microb Pathog.

[35] Hamza EH, El-Shawadfy AM, Allam AA, Hassanein WA (2023). Study on pyoverdine and biofilm production with detection of LasR gene in MDR Pseudomonas aeruginosa. Saudi J Biol Sci.

[36] Alamu J, Kakithakara L, Venkatesan B, Thulukanam J (2022). Correlation of phenotypic and genotypic virulence markers, antimicrobial susceptibility pattern, and outcome of Pseudomonas aeruginosa sepsis infection. Microb Pathog.

[37] Huang H, Shao X, Xie Y, Wang T, Zhang Y, Wang X (2019). An integrated genomic regulatory network of virulence-related transcriptional factors in Pseudomonas aeruginosa. Nat Commun.

[38] Gheorghita AA, Li YE, Kitova EN, Bui DT, Pfoh R, Low KE (2022). Structure of the AlgKX modification and secretion complex required for alginate production and biofilm attachment in Pseudomonas aeruginosa. Nat Commun.

[39] Fortuna A, Collalto D, Schiaffi V, Pastore V, Visca P, Ascenzioni F (2022). The Pseudomonas aeruginosa DksA1 protein is involved in H2O2 tolerance and within-macrophages survival and can be replaced by DksA2. Sci Rep.

[40] Wang T, Du X, Ji L, Han Y, Dang J, Wen J (2021). *Pseudomonas aeruginosa* T6SS-mediated molybdate transport contributes to bacterial competition during anaerobiosis. Cell Rep.

[41] Nolan LM, Cain AK, Clamens T, Furniss RCD, Manoli E, Sainz-Polo MA (2021). Identification of Tse8 as a Type VI secretion system toxin from Pseudomonas aeruginosa that targets the bacterial transamidosome to inhibit protein synthesis in prey cells. Nat Microbiol.

[42] Lujan AM, Paterson S, Hesse E, Sommer LM, Marvig RL, Sharma MD (2022). Polymicrobial infections can select against Pseudomonas aeruginosa mutators because of quorum-sensing trade-offs. Nat Ecol Evol.

[43] Chung H, Merakou C, Schaefers MM, Flett KB, Martini S, Lu R (2022). Rapid expansion and extinction of antibiotic resistance mutations during treatment of acute bacterial respiratory infections. Nat Commun.

[44] Valentin JDP, Straub H, Pietsch F, Lemare M, Ahrens CH, Schreiber F (2022). Role of the flagellar hook in the structural development and antibiotic tolerance of Pseudomonas aeruginosa biofilms. ISME J.

[45] Lichtenberg M, Line L, Schrameyer V, Jakobsen TH, Rybtke ML, Toyofuku M, et al. Nitric-oxide-driven oxygen release in anoxic *Pseudomonas aeruginosa*. iScience. 2021;24(12). 10.1016/j.isci.2021.103404. 10.1016/j.isci.2021.103404 PMC8608891 34849468

[46] Kragh KN, Alhede M, Jensen PO, Moser C, Scheike T, Jacobsen CS (2014). Polymorphonuclear leukocytes restrict growth of Pseudomonas aeruginosa in the lungs of cystic fibrosis patients. Infect Immun.

[47] La Rosa R, Rossi E, Feist AM, Johansen HK, Molin S (2021). Compensatory evolution of Pseudomonas aeruginosa’s slow growth phenotype suggests mechanisms of adaptation in cystic fibrosis. Nat Commun.

[48] Peña JM, Prezioso SM, McFarland KA, Kambara TK, Ramsey KM, Deighan P (2021). Control of a programmed cell death pathway in Pseudomonas aeruginosa by an antiterminator. Nat Commun.

[49] Gdaniec BG, Bonini F, Prodon F, Braschler T, Köhler T, van Delden C. *Pseudomonas aeruginosa* rhamnolipid micelles deliver toxic metabolites and antibiotics into *Staphylococcus aureus*. iScience. 2022;25(1). 10.1016/j.isci.2021.103669. 10.1016/j.isci.2021.103669 PMC8741607 35028539

[50] Gomes-Fernandes M, Gomez A-C, Bravo M, Huedo P, Coves X, Prat-Aymerich C (2022). Strain-specific interspecies interactions between co-isolated pairs of Staphylococcus aureus and Pseudomonas aeruginosa from patients with tracheobronchitis or bronchial colonization. Sci Rep.

[51] Lalsiamthara J, Aballay A (2022). The gut efflux pump MRP-1 exports oxidized glutathione as a danger signal that stimulates behavioral immunity and aversive learning. Commun Biol.

[52] Wang C, Ye Q, Jiang A, Zhang J, Shang Y, Li F (2022). Pseudomonas aeruginosa detection using conventional PCR and quantitative real-time PCR based on species-specific novel gene targets identified by pangenome analysis. Front Microbiol.

[53] He Y, Zhao H, Liu Y, Zhou H (2021). Specific and rapid reverse assaying protocol for detection and antimicrobial susceptibility testing of Pseudomonas aeruginosa based on dual molecular recognition. Sci Rep.

[54] Rydzak T, Groves RA, Zhang R, Aburashed R, Pushpker R, Mapar M (2022). Metabolic preference assay for rapid diagnosis of bloodstream infections. Nat Commun.

[55] Ongwae GM, Chordia MD, Cawley JL, Dalesandro BE, Wittenberg NJ, Pires MM (2022). Targeting of Pseudomonas aeruginosa cell surface via GP12, an Escherichia coli specific bacteriophage protein. Sci Rep.

[56] Cernooka E, Rumnieks J, Zrelovs N, Tars K, Kazaks A (2022). Diversity of the lysozyme fold: structure of the catalytic domain from an unusual endolysin encoded by phage Enc34. Sci Rep.

[57] Li N, Shen M, Liu J, Zhang L, Wang H, Xu Y (2021). Multiplexed detection of respiratory pathogens with a portable analyzer in a “raw-sample-in and answer-out” manner. Microsyst Nanoeng.

[58] Mogyoródi B, Csékó AB, Hermann C, Gál J, Iványi ZD (2022). Ceftolozane/tazobactam versus colistin in the treatment of ventilator-associated pneumonia due to extensively drug-resistant Pseudomonas aeruginosa. Sci Rep.

[59] Montero MM, Domene-Ochoa S, Lopez-Causape C, Luque S, Sorli L, Campillo N (2021). Impact of ceftolozane/tazobactam concentrations in continuous infusion against extensively drug-resistant Pseudomonas aeruginosa isolates in a hollow-fiber infection model. Sci Rep.

[60] Bitar I, Salloum T, Merhi G, Hrabak J, Araj GF, Tokajian S (2022). Genomic characterization of mutli-drug resistant Pseudomonas aeruginosa clinical isolates: evaluation and determination of ceftolozane/tazobactam activity and resistance mechanisms. Front Cell Infect Microbiol.

[61] Ciecholewska-Juśko D, Żywicka A, Junka A, Woroszyło M, Wardach M, Chodaczek G (2022). The effects of rotating magnetic field and antiseptic on in vitro pathogenic biofilm and its milieu. Sci Rep.

[62] Eivazzadeh-Keihan R, Alimirzaloo F, Aghamirza Moghim Aliabadi H, Bahojb Noruzi E, Akbarzadeh AR, Maleki A (2022). Functionalized graphene oxide nanosheets with folic acid and silk fibroin as a novel nanobiocomposite for biomedical applications. Sci Rep.

[63] Wali N, Shabbir A, Wajid N, Abbas N, Naqvi SZH (2022). Synergistic efficacy of colistin and silver nanoparticles impregnated human amniotic membrane in a burn wound infected rat model. Sci Rep.

[64] Paškevičius Š, Dapkutė V, Misiūnas A, Balzaris M, Thommes P, Sattar A (2022). Chimeric bacteriocin S5-PmnH engineered by domain swapping efficiently controls Pseudomonas aeruginosa infection in murine keratitis and lung models. Sci Rep.

[65] Li Y, Zhang L-Y, Zhang C, Zhang Z-R, Liu L (2022). Bioinspired antifouling Fe-based amorphous coating via killing-resisting dual surface modifications. Sci Rep.

[66] Bodede O, Kuali M, Prinsloo G, Moodley R, Govinden R (2022). Anti-Pseudomonas aeruginosa activity of a C16-terpene dilactone isolated from the endophytic fungus Neofusicoccum luteum of Kigelia africana (Lam.). Sci Rep.

[67] Bondareva NE, Soloveva AV, Sheremet AB, Koroleva EA, Kapotina LN, Morgunova EY (2022). Preventative treatment with Fluorothiazinon suppressed Acinetobacter baumannii-associated septicemia in mice. J Antibiot.

[68] Morrison KD, Martin KA, Wimpenny JB, Loots GG (2022). Synthetic antibacterial minerals: harnessing a natural geochemical reaction to combat antibiotic resistance. Sci Rep.

[69] Sripahco T, Khruengsai S, Charoensup R, Tovaranonte J, Pripdeevech P (2022). Chemical composition, antioxidant, and antimicrobial activity of Elsholtzia beddomei C. B. Clarke ex Hook. f. essential oil. Sci Rep.

[70] Mohan A, Al-Sayah MH, Ahmed A, El-Kadri OM (2022). Triazine-based porous organic polymers for reversible capture of iodine and utilization in antibacterial application. Sci Rep.

[71] Mendes CR, Dilarri G, Forsan CF, Sapata VDMR, Lopes PRM, de Moraes PB (2022). Antibacterial action and target mechanisms of zinc oxide nanoparticles against bacterial pathogens. Sci Rep.

[72] Ralte L, Khiangte L, Thangjam NM, Kumar A, Singh YT (2022). GC-MS and molecular docking analyses of phytochemicals from the underutilized plant, Parkia timoriana revealed candidate anti-cancerous and anti-inflammatory agents. Sci Rep.

[73] Guerrini M, d’Agostino S, Grepioni F, Braga D, Lekhan A, Turner RJ (2022). Antimicrobial activity of supramolecular salts of gallium(III) and proflavine and the intriguing case of a trioxalate complex. Sci Rep.

[74] Vieira D, Angel SN, Honjol Y, Masse M, Gruenheid S, Harvey EJ (2022). Engineering surgical stitches to prevent bacterial infection. Sci Rep.

[75] Chandrasekharan S, Chinnasamy G, Bhatnagar S (2022). Sustainable phyto-fabrication of silver nanoparticles using Gmelina arborea exhibit antimicrobial and biofilm inhibition activity. Sci Rep.

[76] Kim DJ, Jung M-Y, Park J-H, Pak H-J, Kim M, Chuck RS (2021). Moxifloxacin releasing intraocular implant based on a cross-linked hyaluronic acid membrane. Sci Rep.

[77] Ramalingam V, Rajaram R, Archunan G, Padmanabhan P, Gulyás B. Structural characterization, antimicrobial, antibiofilm, antioxidant, anticancer and acute toxicity properties of N-(2-hydroxyphenyl)-2-phenazinamine from Nocardiopsis exhalans (KP149558). 2022;12. 10.3389/fcimb.2022.794338. 10.3389/fcimb.2022.794338 PMC9161293 35663469

[78] Leus IV, Weeks JW, Bonifay V, Shen Y, Yang L, Cooper CJ (2022). Property space mapping of Pseudomonas aeruginosa permeability to small molecules. Sci Rep.

[79] Elekhnawy E, Negm WA, El-Aasr M, Kamer AA, Alqarni M, Batiha GE-S (2022). Histological assessment, anti-quorum sensing, and anti-biofilm activities of Dioon spinulosum extract: in vitro and in vivo approach. Sci Rep.

[80] Chadha J, Ravi, Singh J, Chhibber S, Harjai K. Gentamicin augments the quorum quenching potential of cinnamaldehyde in vitro and protects Caenorhabditis elegans from Pseudomonas aeruginosa infection. 2022;12. 10.3389/fcimb.2022.899566. 10.3389/fcimb.2022.899566 PMC9240785 35782125

[81] Dawoud NTA, El-Fakharany EM, Abdallah AE, El-Gendi H, Lotfy DR (2022). Synthesis, and docking studies of novel heterocycles incorporating the indazolylthiazole moiety as antimicrobial and anticancer agents. Sci Rep.

[82] Singh VK, Almpani M, Maura D, Kitao T, Ferrari L, Fontana S (2022). Tackling recalcitrant Pseudomonas aeruginosa infections in critical illness via anti-virulence monotherapy. Nat Commun.

[83] Blanco-Cabra N, Movellan J, Marradi M, Gracia R, Salvador C, Dupin D (2022). Neutralization of ionic interactions by dextran-based single-chain nanoparticles improves tobramycin diffusion into a mature biofilm. NPJ Biofilms Microbiomes.

[84] Nikolaou A, Felipe-Sotelo M, Dorey R, Gutierrez-Merino J, Carta D (2022). Silver-doped phosphate coacervates to inhibit pathogenic bacteria associated with wound infections: an in vitro study. Sci Rep.

[85] Shukla A, Shukla G, Parmar P, Patel B, Goswami D, Saraf M (2021). Exemplifying the next generation of antibiotic susceptibility intensifiers of phytochemicals by LasR-mediated quorum sensing inhibition. Sci Rep.

[86] Tan Lim AM, Oyong GG, Tan MCS, Chang Shen C, Ragasa CY, Cabrera EC. Quorum quenching activity of *Andrographis paniculata* (Burm f.) Nees andrographolide compounds on metallo-β-lactamase--producing clinical isolates of *Pseudomonas aeruginosa* PA22 and PA247 and their effect on *lasR* gene expression. Heliyon. 2021;7(5). 10.1016/j.heliyon.2021.e07002. 10.1016/j.heliyon.2021.e07002 PMC8131311 34027192

[87] Ducki S, Hadfield JA, Lawrence NJ, Zhang X, McGown AT (1995). Isolation of paeonol from Arisaema erubescens. Planta Med.

[88] Chen B, Ning M, Yang G (2012). Effect of paeonol on antioxidant and immune regulatory activity in hepatocellular carcinoma rats. Molecules (Basel, Switzerland).

[89] Lou Y, Wang C, Tang Q, Zheng W, Feng Z, Yu X (2017). Paeonol inhibits IL-1beta-induced inflammation via PI3K/Akt/NF-kappaB pathways: in vivo and vitro studies. Inflammation.

[90] Qian W, Li X, Yang M, Mao G (2021). Antibacterial and anti-biofilm activities of paeonol against Klebsiella pneumoniae and Enterobacter cloacae. Biofouling.

[91] Zeng Q, Fu Y, Yang M, Wang T, Wang Y, Lv S (2022). Effect of paeonol against bacterial growth, biofilm formation and dispersal of Staphylococcus aureus and Listeria monocytogenes in vitro. Biofouling.

[92] Tang H, Yang D, Zhu L, Shi F, Ye G, Guo H (2022). Paeonol interferes with quorum-sensing in Pseudomonas aeruginosa and modulates inflammatory responses in vitro and in vivo. Front Immunol.

[93] Yang D, Hao S, Zhao L, Shi F, Ye G, Zou Y (2021). Paeonol attenuates quorum-sensing regulated virulence and biofilm formation in Pseudomonas aeruginosa. Front Microbiol.

[94] Singh P, Mijakovic I (2022). Green synthesis and antibacterial applications of gold and silver nanoparticles from Ligustrum vulgare berries. Sci Rep.

[95] Jeyanthi V, Velusamy P, Kumar GV, Kiruba K (2021). Effect of naturally isolated hydroquinone in disturbing the cell membrane integrity of Pseudomonas aeruginosa MTCC 741 and Staphylococcus aureus MTCC 740. Heliyon.

[96] El Feky SE, Abd El Hafez MSM, Abd El Moneim NA, Ibrahim HAH, Okbah MA, Ata A (2022). Cytotoxic and antimicrobial activities of two new sesquiterpenoids from red sea brittle star Ophiocoma dentata. Sci Rep.

[97] Foell D, Kucharzik T, Kraft M, Vogl T, Sorg C, Domschke W (2003). Neutrophil derived human S100A12 (EN-RAGE) is strongly expressed during chronic active inflammatory bowel disease. Gut.

[98] Mishra P, Ch S, Hong SJ, Biswas S, Roy S (2022). Antimicrobial peptide S100A12 (calgranulin C) inhibits growth, biofilm formation, pyoverdine secretion and suppresses type VI secretion system in Pseudomonas aeruginosa. Microb Pathog.

[99] Nordstrom HR, Evans DR, Finney AG, Westbrook KJ, Zamora PF, Hofstaedter CE, et al. Genomic characterization of lytic bacteriophages targeting genetically diverse *Pseudomonas aeruginosa* clinical isolates. iScience. 2022;25(6). 10.1016/j.isci.2022.104372. 10.1016/j.isci.2022.104372 PMC9127202 35620437

[100] Hendrix H, Zimmermann-Kogadeeva M, Zimmermann M, Sauer U, De Smet J, Muchez L, et al. Metabolic reprogramming of *Pseudomonas aeruginosa* by phage-based quorum sensing modulation. Cell Rep. 2022;38(7). 10.1016/j.celrep.2022.110372. 10.1016/j.celrep.2022.110372 35172131

[101] De Smet J, Wagemans J, Boon M, Ceyssens P-J, Voet M, Noben J-P, et al. The bacteriophage LUZ24 “Igy” peptide inhibits the *Pseudomonas* DNA gyrase. Cell Rep. 2021;36(8). 10.1016/j.celrep.2021.109567. 10.1016/j.celrep.2021.109567 34433028

[102] Di Pietro C, Öz HH, Zhang P-x, Cheng E-c, Martis V, Bonfield TL, et al. Recruitment of monocytes primed to express heme oxygenase-1 ameliorates pathological lung inflammation in cystic fibrosis. Exp Mol Med. 2022. 10.1038/s12276-022-00770-8. 10.1038/s12276-022-00770-8 PMC9166813 35581352

[103] Hochvaldová L, Panáček D, Válková L, Prucek R, Kohlová V, Večeřová R (2022). Restoration of antibacterial activity of inactive antibiotics via combined treatment with a cyanographene/Ag nanohybrid. Sci Rep.

[104] Xu C, Liu C, Chen K, Zeng P, Chan EWC, Chen S (2022). Otilonium bromide boosts antimicrobial activities of colistin against Gram-negative pathogens and their persisters. Commun Biol.

[105] Roberts KD, Zhu Y, Azad MAK, Han M-L, Wang J, Wang L (2022). A synthetic lipopeptide targeting top-priority multidrug-resistant Gram-negative pathogens. Nat Commun.

[106] Henninot A, Collins JC, Nuss JM (2018). The current state of peptide drug discovery: back to the future?. J Med Chem.

[107] Xiang W, Clemenza P, Klousnitzer J, Chen J, Qin W, Tristram-Nagle S, et al. Rational framework for the design of Trp- and Arg-Rich peptide antibiotics against multidrug-resistant bacteria. 2022;13. 10.3389/fmicb.2022.889791. 10.3389/fmicb.2022.889791 PMC9186412 35694289

[108] Lai X, Han M-L, Ding Y, Chow SH, Le Brun AP, Wu C-M (2022). A polytherapy based approach to combat antimicrobial resistance using cubosomes. Nat Commun.

[109] Stokniene J, Varache M, Rye PD, Hill KE, Thomas DW, Ferguson EL (2022). Alginate oligosaccharides enhance diffusion and activity of colistin in a mucin-rich environment. Sci Rep.

[110] Huang JX, Blaskovich MAT, Pelingon R, Ramu S, Kavanagh A, Elliott AG (2015). Mucin binding reduces colistin antimicrobial activity. Antimicrob Agents Chemother.

[111] O’Brien TJ, Figueroa W, Welch M (2022). Decreased efficacy of antimicrobial agents in a polymicrobial environment. ISME J.

[112] Abdelraheem WM, Refaie MMM, Yousef RKM, Abd El Fatah AS, Mousa YM, Rashwan R. Assessment of antibacterial and anti-biofilm effects of vitamin C against Pseudomonas aeruginosa clinical isolates. 2022;13. 10.3389/fmicb.2022.847449. 10.3389/fmicb.2022.847449 PMC9163820 35668756

[113] Sundaran S, Kok L-C, Chang H-Y (2022). Combination effect of epsilon-poly-L-lysine and antibiotics against common bacterial pathogens. J Antibiot.

[114] Lee HR, Lee YS, You YS, Huh JY, Kim K, Hong YC (2022). Antimicrobial effects of microwave plasma-activated water with skin protective effect for novel disinfectants in pandemic era. Sci Rep.

[115] Ajish C, Yang S, Kumar SD, Kim EY, Min HJ, Lee CW (2022). A novel hybrid peptide composed of LfcinB6 and KR-12-a4 with enhanced antimicrobial, anti-inflammatory and anti-biofilm activities. Sci Rep.

[116] Yamamoto K, Yamamoto N, Ayukawa S, Yasutake Y, Ishiya K, Nakashima N (2022). Scaffold size-dependent effect on the enhanced uptake of antibiotics and other compounds by Escherichia coli and Pseudomonas aeruginosa. Sci Rep.

[117] Kong L, Vijayakrishnan B, Kowarik M, Park J, Zakharova AN, Neiwert L (2016). An antibacterial vaccination strategy based on a glycoconjugate containing the core lipopolysaccharide tetrasaccharide Hep2Kdo2. Nat Chem.

[118] Hoggarth A, Weaver A, Pu Q, Huang T, Schettler J, Chen F (2019). Mechanistic research holds promise for bacterial vaccines and phage therapies for Pseudomonas aeruginosa. Drug Des Dev Ther.

[119] Finke M, Duchêne M, Eckhardt A, Domdey H, Specht BUV (1990). Protection against experimental Pseudomonas aeruginosa infection by recombinant P. aeruginosa lipoprotein I expressed in Escherichia coli. Infect Immun.

[120] Low KE, Howell PL (2018). Gram-negative synthase-dependent exopolysaccharide biosynthetic machines. Curr Opin Struct Biol.

[121] Sawa T, Wiener-Kronish JP (2004). A therapeutic strategy against the shared virulence mechanism utilized by both Yersinia pestis and Pseudomonas aeruginosa. Anesthesiol Clin North America.

[122] De Tavernier E, Detalle L, Morizzo E, Roobrouck A, De Taeye S, Rieger M (2016). High throughput combinatorial formatting of PcrV nanobodies for efficient potency improvement*. J Biol Chem.

[123] Jiang M, Yao J, Feng G (2014). Protective effect of DNA vaccine encoding Pseudomonas exotoxin A and PcrV against acute pulmonary P. aeruginosa infection. PLoS One.

[124] Sabzehali F, Rahimi H, Goudarzi H, Goudarzi M, Yoosefi Izad MH, Salimi Chirani A (2021). Functional engineering of OprF-OprI-PopB as a chimeric immunogen and its cross-protective evaluation with GM-CSF against Pseudomonas aeruginosa: a comprehensive immunoinformatics evaluation. Inform Med Unlocked.

[125] Maleki M, Azimi S, Salouti M (2022). Protective effect of two new nanovaccines against Pseudomonas aeruginosa based on LPS and OPS: a comparison study. Immunobiology.

[126] Guo Z, Zhu Y, Du G, Qin M, He C, He P (2022). Rapid development of a subunit nano-vaccine against drug-resistant Pseudomonas aeruginosa with effective cross-protection. Nano Today.

[127] Zhang L, Zhang Y, Hua Q, Xu T, Liu J, Zhu Y (2022). Promoter-controlled synthesis and antigenic evaluation of mannuronic acid alginate glycans of Pseudomonas aeruginosa. Org Lett.

[128] Asadi Karam MR, Badmasti F, Ahmadi K, Habibi M (2022). Vaccination of mice with hybrid protein containing Exotoxin S and PcrV with adjuvants alum and MPL protects Pseudomonas aeruginosa infections. Sci Rep.

[129] Ma C, Ma X, Jiang B, Pan H, Liao X, Zhang L (2021). A novel inactivated whole-cell Pseudomonas aeruginosa vaccine that acts through the cGAS-STING pathway. Signal Transduct Target Ther.

[130] Zare Banadkoki E, Rasooli I, Ghazanfari T, Siadat SD, Shafiee Ardestani M, Owlia P (2022). Pseudomonas aeruginosa PAO1 outer membrane vesicles-diphtheria toxoid conjugate as a vaccine candidate in a murine burn model. Sci Rep.

[131] Kaparakis-Liaskos M, Ferrero RL (2015). Immune modulation by bacterial outer membrane vesicles. Nat Rev Immunol.

[132] Gnopo YMD, Watkins HC, Stevenson TC, DeLisa MP, Putnam D (2017). Designer outer membrane vesicles as immunomodulatory systems – reprogramming bacteria for vaccine delivery. Adv Drug Deliv Rev.

[133] Rahbar MR, Mubarak SMH, Hessami A, Khalesi B, Pourzardosht N, Khalili S (2022). A unique antigen against SARS-CoV-2, Acinetobacter baumannii, and Pseudomonas aeruginosa. Sci Rep.

[134] Gao C, Yang F, Wang Y, Liao Y, Zhang J, Zeng H (2017). Vaccination with a recombinant OprL fragment induces a Th17 response and confers serotype-independent protection against Pseudomonas aeruginosa infection in mice. Clin Immunol.

[135] Gomi R, Sharma A, Wu W, Sung B, Worgall S (2017). Post-exposure immunization by capsid-modified AdC7 vector expressing Pseudomonas aeruginosa OprF clears P. aeruginosa respiratory infection. Vaccine.

[136] Gong Q, Li Y, Zhai W, Niu M (2023). Immune responses and protective efficacy of a trivalent combination DNA vaccine based on oprL, oprF and flgE genes of Pseudomonas aeruginosa. Vet Med.

[137] Gong Q, Ruan M, Niu M, Qin C (2021). Immune efficacy of different immunization doses of divalent combination DNA vaccine pOPRL+pOPRF of *Pseudomonas aeruginosa*. J Vet Med Sci.

[138] Blackwood CB, Mateu-Borrás M, Sen-Kilic E, Pyles GM, Miller SJ, Weaver KL (2022). Bordetella pertussis whole cell immunization protects against Pseudomonas aeruginosa infections. NPJ Vaccines.

[139] Dehnavi M, Haghighat S, Yazdi MH, Mahdavi M (2023). Glucomannan as a polysaccharide adjuvant improved immune responses against Staphylococcus aureus: potency and efficacy studies. Microb Pathog.

[140] Terra VS, Mills DC, Yates LE, Abouelhadid S, Cuccui J, Wren BW (2012). Recent developments in bacterial protein glycan coupling technology and glycoconjugate vaccine design. J Med Microbiol.

[141] Avci F, Berti F, Dull P, Hennessey J, Pavliak V, Prasad AK, et al. Glycoconjugates: what it would take to master these well-known yet little-understood immunogens for vaccine development. 2019;4(5):e00520–19. 10.1128/mSphere.00520-19. 10.1128/mSphere.00520-19 PMC6763769 31554723

[142] Pöllabauer EM, Petermann R, Ehrlich HJ (2009). The influence of carrier protein on the immunogenicity of simultaneously administered conjugate vaccines in infants. Vaccine.

[143] Tontini M, Romano MR, Proietti D, Balducci E, Micoli F, Balocchi C (2016). Preclinical studies on new proteins as carrier for glycoconjugate vaccines. Vaccine.

[144] Lee JW, Parlane NA, Wedlock DN, Rehm BHA (2017). Bioengineering a bacterial pathogen to assemble its own particulate vaccine capable of inducing cellular immunity. Sci Rep.

[145] Jurcisek JA, Hofer LK, Goodman SD, Bakaletz LO (2022). Monoclonal antibodies that target extracellular DNABII proteins or the type IV pilus of nontypeable Haemophilus influenzae (NTHI) worked additively to disrupt 2-genera biofilms. Biofilm.

[146] Warr GW, Magor KE, Higgins DA (1995). IgY: clues to the origins of modern antibodies. Immunol Today.

[147] Tini M, Jewell UR, Camenisch G, Chilov D, Gassmann M (2002). Generation and application of chicken egg-yolk antibodies. Comp Biochem Physiol A: Mol Integr Physiol.

[148] Ranjbar M, Behrouz B, Norouzi F, Mousavi Gargari SL (2019). Anti-PcrV IgY antibodies protect against Pseudomonas aeruginosa infection in both acute pneumonia and burn wound models. Mol Immunol.

[149] Sanches RF, dos Santos Ferraro ACN, Marroni FEC, Venancio EJ (2022). Synergistic activity between beta-lactams and igy antibodies against Pseudomonas aeruginosa in vitro. Mol Immunol.

[150] Patel A, DiGiandomenico A, Keller AE, Smith TRF, Park DH, Ramos S (2017). An engineered bispecific DNA-encoded IgG antibody protects against Pseudomonas aeruginosa in a pneumonia challenge model. Nat Commun.

[151] Chastre J, François B, Bourgeois M, Komnos A, Ferrer R, Rahav G (2022). Safety, efficacy, and pharmacokinetics of gremubamab (MEDI3902), an anti-Pseudomonas aeruginosa bispecific human monoclonal antibody, in P. aeruginosa-colonised, mechanically ventilated intensive care unit patients: a randomised controlled trial. Crit Care.

[152] Zamani K, Irajian G, Zahedi Bialvaei A, Zahraei Salehi T, Khormali M, Vosough A (2022). Passive immunization with anti- chimeric protein PilQ/PilA –DSL region IgY does not protect against mortality associated with Pseudomonas aeruginosa sepsis in a rabbit model. Mol Immunol.

[153] Ahmadi TS, Mousavi Gargari SL, Talei D (2021). Anti-flagellin IgY antibodies protect against Pseudomonas aeruginosa infection in both acute pneumonia and burn wound murine models in a non-type-specific mode. Mol Immunol.

